# Sleep Fainting: A Neurocardiogenic Entity

**DOI:** 10.7759/cureus.3751

**Published:** 2018-12-18

**Authors:** Hunaina Shahab, Ambreen A Sonawalla, Maryam Khan, Aman Qureshi, Aamir H Khan

**Affiliations:** 1 Cardiology, Aga Khan University Hospital, Karachi, PAK; 2 Pediatrics, University of Arkansas for Medical Sciences, Little Rock, USA; 3 Internal Medicine, Ziauddin University, Karachi, PAK; 4 Miscellaneous, Ziauddin University, Karachi, PAK

**Keywords:** syncope, neurocardiogenic syncope, sleep syncope

## Abstract

Fainting is a common clinical presentation, with vagally mediated (neurocardiogenic) causes being the most common for syncope presentation to the emergency room, and for hospital admissions. Classic teaching is that upright posture is a prerequisite for vagally mediated syncope (VMS) and that syncope in the supine position has more sinister causes. We present five patients, three males and two females, with a mean age of 44.4 (range 29–67) years, who presented with VMS in the supine position (sleep fainting). Four patients also had a history of classic upright syncope. Based on their clinical features and thorough investigations, we excluded other causes of loss of consciousness and diagnosed these patients to be having VMS in the supine position (sleep fainting). We further describe the management and follow-up of these patients. Sleep fainting/syncope is a new entity and has to be recognized for appropriate management. A diagnosis can be established if there is clinical suspicion, preserved left ventricular function without evidence of coronary artery disease, no high-risk electrocardiographic evidence of pre-excitation, long or short QT syndrome, Brugada syndrome or arrhythmogenic right ventricular dysplasia, and normal neurological work-up.

## Introduction

Syncope is defined as a transient loss of consciousness due to cerebral hypoperfusion with recovery being spontaneous and instantaneous [1-2]. It is a common ailment that forms 3% of emergency admissions and 1% of general hospital admissions [3]. Of all the causes of syncope, neurocardiogenic, or vagally mediated syncope (VMS) is the most common [4], with syncope secondary to cardiac causes having the worst prognosis [5]. The upright posture leads to pooling of blood in the legs and the visceral circulation, reducing cardiac preload and output, thereby activating the sympathetic and inhibiting the parasympathetic system [6-7]. Contraction of an inadequately filled ventricle leads to stimulation of cardiac mechanoreceptors which leads to decreased sympathetic and increased parasympathetic response through the brainstem causing bradycardia, hypotension, and syncope [8-9]. This is classically noted in the upright posture, being rare in the supine posture due to adequate maintenance of cerebral perfusion due to gravity [10].

However, recent literature now supports the hypothesis that both vagally mediated hypotension and cardioinhibition can be noted while in a supine position. Several reports of supine loss of consciousness, which was determined to be vagal in mechanism, have been reported in the literature [1,11-13], and in 2006, a study named this phenomenon “sleep syncope” or “sleep fainting” and suggested it as a new clinical entity [14]. Furthermore, this phenomenon is not an uncommon observation in hospital settings in which the patient is supine, such as while venepuncture is performed and vagal symptoms are noted [15]. Similarly, during anesthesia when the patient is unconscious, similar vagal slowing is noted. This is observed especially during pelvic and ophthalmic surgeries [16].

We report five cases where the patients experienced unexplained supine fainting – a brief unarousable state during sleep or a loss of consciousness while awake and supine. We describe the details and similarities in the clinical accounts.

## Case presentation

Case 1

A 29-year-old woman with a history of asthma (on intermittent inhaler use) came in with one episode of unarousable loss of consciousness. She was noted to have irregular breathing that alarmed the mother who was sleeping next to her. She called for help and the brother then tried to wake her up but she was not arousable. According to the account, she was sweating profusely and had labored breathing. She spontaneously started breathing normally and started coming around. At this point, she felt very weak, experienced nausea and vomiting, and felt like her “stomach was upset”. Her post-event recovery was less than five minutes. She was brought to the hospital. She is a nurse by profession and was doing her normal ward duties and reports no remarkable event in her history that would have suggested volume loss or prolonged hemodynamic stress. Her examination was unremarkable and there was no postural drop in blood pressure (BP). She was admitted under neurology; her blood and electrolyte work-up was normal and she underwent a complete neurological work-up. She was then referred to cardiology at which point it was noted that she had pre-excitation on the electrocardiogram (ECG). She was taken to the catheterization lab where she underwent an electrophysiological (EP) study that induced an orthodromic atrioventricular tachycardia. Atrial fibrillation was induced through rapid atrial pacing but could not be sustained and no ventricular arrhythmias were inducible without or with dobutamine provocation. Ablation of a left posteroseptal pathway was carried out. The earliest ventricular signal was tracked during tachycardia and ventricular pacing but ablation at the earliest position suppressed accessory pathway conduction only to recur every time. After extensively mapping the right side and the coronary sinus, the ablation was stopped. She was put on flecainide and calcium channel blockers and was discharged. After a few weeks, she had a similar episode and was brought to the hospital. She then underwent a head-up tilt (HUT) test on suspicion of vasovagal mechanism of syncope, which came out positive with nitrate provocation. Her post-ictal phase was reported by her to be an exact replication of her clinical symptoms. The flecainide and calcium channel blockers were discontinued as the mechanism of her fainting was identified to be vasovagal and not a cardiac arrhythmia. She was prescribed hydration, counter-maneuvers (which she practiced inconsistently), and preventive measures. Since the last episode, she has done well but experiences recurrences.

Case 2

A 33-year-old woman, married with four children, presented to the clinic with a history of four episodes of unresponsiveness. Two were in an upright posture in the morning hours while she was preparing breakfast for her children. The other two were while she was sleeping and her husband discovered that her breathing pattern had become irregular. He tried to arouse her but could not do so. He reported that she was sweating profusely. She recovered in less than five minutes but was too weak to move and had abdominal cramps and a desire to defecate. The first such episode was short and she had not sought medical help. After the second episode, she was seen at a cardiology tertiary care center. Her exam and basic laboratory work were unremarkable. She did not have a postural drop in BP. Her ECG showed normal sinus rhythm with frequent ventricular ectopy of the right ventricular outflow tract origin. An echocardiogram revealed structurally normal heart with preserved left and right ventricular functions. In view of ventricular tachycardia as a cause of her syncope, she was offered an EP study and ablation. She was brought to the EP laboratory, where her rhythm was normal sinus rhythm and no ectopy could be induced despite isoprenaline provocation. She was then discharged on calcium channel blockers. At the time of her visit, she was doing well and had no ventricular ectopy. She underwent a HUT test which was positive with nitrate provocation. She stated that her symptoms on the HUT table were consistent with the ones that she had been experiencing during the episodes. She was prescribed hydration, counter-maneuvers and preventive measures. Since the last episode, she has not had any recurrence.

Case 3

A 43-year-old man who is a hospital worker came to the clinic with three successive syncopal attacks, all in the morning hours. One was noted while he was supine and got up from sleep. He was sweating profusely according to him and it was witnessed by his wife. He felt nauseous and had an abdominal gripe and passed out while in bed. He recovered spontaneously after a few minutes while his wife tried to revive him. On examination, he did not have a postural drop in BP. His ECG was within normal limits and an echocardiogram was conducted which was normal. His blood and biochemical work-up were normal. He underwent a HUT test which was positive with nitrate provocation. He was reassured and sent home with advice to remain hydrated, apply counter-maneuvers and exercise. After his clinic visit, he had another episode of supine fainting and this time his wife raised his legs and he reports that despite experiencing the same symptoms, the duration of the episode was abbreviated. He has done well since the institution of regular hydration and exercising the counter-maneuvers, though he still experiences recurrent episodes.

Case 4

A 51-year-old man presented to us with three episodes of loss of consciousness. He had an aortic valve replacement with a metallic valve a few years ago and did not have any other cardiac risk factors and was fairly active. He took warfarin and his international normalized ratio (INR) was within therapeutic range. The day of the admission, he has been out the whole day and had not eaten or taken the usual amount of liquids; the initiating event took place at a funeral, where he felt nauseous and dizzy. This was in the evening time. At night, he went to operate the water pump and had an unheralded fall and recovered spontaneously. He was brought up to his room where he lay with head propped up. At that time, he felt a griping pain in the abdomen and experienced nausea and passed out. According to the wife, he came around with sprinkling of water. He complained of worsening of the gripe and felt like vomiting. By this time he was totally lying flat in bed. The wife witnessed his eyes rolling up and he had myoclonic jerks. He came around in a couple of minutes and was rushed to the hospital. On examination, he has normal range pulse and BP, while the rest of the exam was unremarkable. His ECG (sinus rhythm with right bundle-branch block), blood work-up and echocardiogram were normal. His ejection fraction (EF) was 55% and the prosthetic aortic valve was functioning normally. His neurological assessment, electroencephalogram (EEG) and scan ruled out a neurologic cause (hemorrhage or thromboembolic event). He then underwent a HUT test, which came out positive at three minutes post nitrate provocation with a cardio-inhibitory response, where the ECG showed severe sinus bradycardia and sinoatrial (SA) exit block. He reproduced and confirmed all the clinical symptoms of an abdominal gripe, nausea, and sweating. He also felt like moving his bowel at the time. He was prescribed hydration, counter-maneuvers and walking. He did not have any recurrence in the follow-up period.

Case 5

A 67-year-old man who was functionally very active, presented with a history of fainting while in bed lying flat. He had similar multiple episodes. The episodes were noted during sleep, where his breathing would become labored and he had difficulty being aroused from sleep. These episodes were during the period when he had “stomach flu”. The five noted episodes were spread over months. The last one was prolonged with longer recovery time, necessitating a hospital visit. He did give a history of upright syncope in his youth. He had a history of hypertension for which he was well controlled on calcium channel blockers and low dose hydrochlorothiazide. On admission, his pulse and BP were in range and there was no postural drop. His ECG, blood work-up and echocardiogram were normal. He also had a 24 hours Holter monitoring, which showed normal diurnal variation and appropriate nocturnal slowing and there was no episode of blocks or pauses. The neurologic clinical assessment and workup were normal for age. He underwent a HUT test, which revealed a sinus arrest with pause (nine seconds) after five minutes of nitrate provocation. He experienced sweating, nausea, and abdominal pain prior to syncope on the HUT test; during his phase, he showed sinus bradycardia. He was prescribed hydration and measures to take care of the trigger in time as well as counter-maneuvers (leg crossing and muscle tensing) and walking. He has been followed-up without recurrence.

## Discussion

Epilepsy tops the list of differential diagnosis, when patients present with syncope during sleep, other causes being sleep paralysis, sleep apnea, hypoglycemia, panic attacks or arrhythmias [12]. Vagally mediated (neurocardiogenic) syncope, or VMS, is usually not considered because classical teaching postulates that VMS occurs in the upright posture, and not in the supine position.

However, our group of patients had very convincing clinical features to suggest a vagal mechanism for the syncope (Table 1). 

**Table 1:** Clinical features, symptoms, and workup of the five patients who presented with syncope BP: blood pressure; AVR: aortic valve regurgitation; LVEF-N: left ventricular ejection fraction-normal; ECG: electrocardiogram; WPW: wolff-parkinson-white; LPS: left posteroseptal; RVOT: right ventricular outflow tract; PVC: premature ventricular contractions; RBBB: right bundle branch block; EP: electrophysiology.

Case #	1	2	3	4	5
Age	29	32	43	51	67
Gender	F	F	M	M	M
Supine fainting	Yes	Yes	Yes	Yes	Yes
Frequency of syncope	2	4	4	3	5
Sweating	Yes	Yes	Yes	Yes	Yes
Abdominal pain	Yes	Yes	Yes	Yes	Yes
Nausea	Yes	Yes	Yes	Yes	Yes
Urge to defecate	No	Yes	No	Yes	No
Postural drop in BP	No	No	No	Not checked	No
Classical vagal syncope	No	Yes	Yes	Yes	Yes
Triggers	-	No	No	No	Yes
Echocardiogram	Normal	Normal	Normal	AVR (LVEF –N)	Normal
HUT test	+	+	+	+	+
Symptoms replicated	Yes	Yes	Yes	Yes	Yes
ECG changes	WPW (LPS)	RVOT PVC	Normal	RBBB	Normal
EP Study and ablation	Ablation failed	No inducible ectopy	Not required	Not required	Not required
Neurological work-up	Negative	Negative	Negative	Negative	Negative
Counter maneuvers	Yes	Yes	Yes	No	Yes
Recurrence after clinical visit	Yes	No	Yes	No	No

The occurrence of vagal symptoms (sweating, abdominal pain, nausea, and defecation urge) before and/or during the syncopal episodes indicates vagal over-activity accompanying the syncope. Further, a positive HUT test in all the patients, with a re-demonstration of the symptoms experienced during their episodes, indicates not only a tendency for VMS in these patients but also that similar mechanisms were responsible for both the supine syncope and the syncope experienced during the HUT test. Other causes for episodic loss of consciousness were also ruled out in these patients. Neurological causes were excluded by referral to a neurologist; adequate work-up and a lack of postural drop in blood pressure in four of the five patients excluded orthostatic hypotension. A cardiac work-up also found no structural heart abnormalities, nor any electrical abnormalities that would explain the syncopal episodes in the patients. There were no high-risk electrocardiographic evidence of pre-excitation, long or short QT syndrome, Brugada syndrome, or arrhythmogenic right ventricular dysplasia.

Although supine vasovagal syncope is an uncommon occurrence, there have been several reports of its kind in the past [1,11-13], and our patient group had many characteristics similar to those in the earlier reports. The mean age of our patients was 44.4 years, with a range of 29 to 67 years. This is comparable to that reported by Krediet et al. (45 years, range 21-72 years) [12] and Khadilkar et al. (35.9 years) [1]. The vagal prodromal symptoms of nausea, sweating and abdominal pain was found in all our patients, whereas the urge to defecate was reported by two of the five (40%). Krediet et al. had reported a very similar pattern with 85%-92% of patients reporting nausea, abdominal pain and the urge to defecate [12]. Khadilkar et al., however, found a different set of symptoms, with visual blurring being the most common (26%), and headache and sweating reported in only 20% and 13.3% respectively. The duration of the loss of consciousness in our patients was less than five minutes, which is similar to that found in the series reported by Krediet et al. (mean 171 seconds) [12]. Four of our five patients (80%) also reported classic vasovagal syncope (in the standing position) at other times. Khadilkar et al. specifically focused on the relationship of body posture with syncope and found that four (80%) of the five patients who experienced supine syncope, also experienced episodes in the standing and sitting positions, and 20% reported episodes while sitting and supine [1]. However, in the series published by Krediet et al., only 69.2% of the patients who experienced supine syncope also reported episodes in an upright position [12]. All our patients underwent the HUT test, without or with provocation, which was positive in all. Khadilkar et al. also reported that the mechanism identified in all their patients who experienced supine syncope was vasovagal [1]. Krediet et al. reported a positive test in only seven out of eleven (63.6%) of their patients. However, the test was not conducted in two of the 13 patients, and the patients who underwent the test were not given pharmacological provocation [12].

All our patients had a normal interictal electroencephalography and no significant finding on neurological work-up. The patients in the series by Khadilkar et al. also had neurological and other cardiac causes ruled out as part of the exclusion criteria [1]. Krediet et al., however, only reported EEG done in seven of the thirteen patients. Six of these had a normal EEG, of which one was fortunately done during the episode [12].

None of our patients reported specific triggers for their supine syncope, and neither did the patients in the series by Krediet et al. Yet, one patient in our group (20%) reported the sight of blood as a trigger for his syncope in the erect position. However, Khadilkar et al. reported that in patients experiencing erect syncope, blood was only a trigger in two of their 111 patients (4.26%), whereas the most common trigger in the erect position was micturition (36.17%). In the supine position, on the other hand, they found that the most common trigger was smell (50%), followed by intense pain (16.6%) [1].

Two other reports in the literature have described specific stimuli for recurrent supine syncope. In one, recurrent supine syncope was found to be due to pain from gastroesophageal reflux, and the syncope episodes resolved on the treatment of the reflux [13]. A second report also found pain to be the triggering factor; however, this time the pain arose from metastatic liver cancer which went undiagnosed for months [11].

Sleep fainting or “sleep syncope” was suggested as a new clinical entity in, 2006, by Jardine et al. and defined as “loss of consciousness in a non-intoxicated adult occurring during the normal hours of sleep (e.g., 10:00 pm to 7:00 am). The patient wakes up feeling faint, often with abdominal symptoms and may briefly lose consciousness in bed or immediately upon standing. There is no tongue biting or post-ictal confusion. There is usually a history of previous daytime vasovagal syncope but the physical examination, ECG and EEG are within normal limits” [14]. Hence, we suggest that all our patients experienced “sleep fainting”, or vasovagal syncope while supine.

The above-mentioned description, however, does not constitute a diagnostic criterion, and in the absence of clearly defined diagnostic criteria to date, diagnosis of sleep fainting is a challenge. It is firstly important to exclude other potentially more serious causes of the loss of consciousness, namely neurological or cardiac causes such as epilepsy, cardiac structural defects, and cardiac arrhythmias. This was accomplished, in our patients, with a detailed history and a complete work-up which included ECG, cardiac echo, an EP study where deemed necessary, EEG, and brain imaging where required. All of these failed to identify a cause for the fainting. Even the findings described in the ECGs were not found to be significant enough to explain the syncope. Although an EEG during the supine syncope would be the most beneficial, a normal interictal EEG along with an adequate history that showed the absence of postictal confusion, tongue biting, and unusual posturing, and occurrence of presyncope with diaphoresis and nausea served as evidence to rule out seizures [17]. To obtain objective evidence of vasovagal syncope occurring during sleep, a polysomnography during the episode would be indispensable. However, due to the sporadic nature of these events, the costs and inconvenience associated with such a monitoring would not be justified.

Vasovagal syncope while supine is not as yet fully explained by known physiological mechanisms. However, several hypotheses have been postulated. Firstly, the increase in centrally-mediated vagal activity during the deeper stages of non-rapid eye movement (REM) sleep may lead to supine vasovagal syncope in individuals with a tendency for excess vagal activity [14]. Secondly, the greater inter-dependence of heart rate and respiration during sleep may allow a decrease in respiration during deep non-REM sleep to cause an exaggerated impairment of venous return as compared to the effect when awake. This could then potentially lead to cerebral hypoperfusion [14].

Evidence for a vagally mediated mechanism of supine syncope has also been lent by a recent series, of 111 patients presenting with syncope, by Khadilkar et al. [1]. It found that in individual patients, the prodromal symptoms for syncope in all positions (standing, sitting, supine) remained the same, as did events during and after the syncopal episodes. This suggested that the sequence of events in the syncopal episode once started, remained the same irrespective of body position, indicating that the vagal activity responsible for erect syncope also occurs in the supine position in a similar fashion [1].

## Conclusions

Sleep fainting is a new entity and has to be recognized for its appropriate management. We conclude that all of the five patients may have sleep fainting (supine vasovagal syncope) as the primary cause of their symptoms since all of them reported symptoms of vagal stimulation, reproduced their symptoms on a HUT test, and had cardiac and neurological work-ups negative for causes of loss of consciousness. There is no defined criterion for its diagnosis, which has to be made on grounds of strong clinical suspicion and exclusion of other neurological and cardiac causes. Counter-maneuvers, though described in the literature to be helpful in such cases, are not effective in all patients. However, reassurance and avoidance of any stimuli that may cause triggers are essential.
